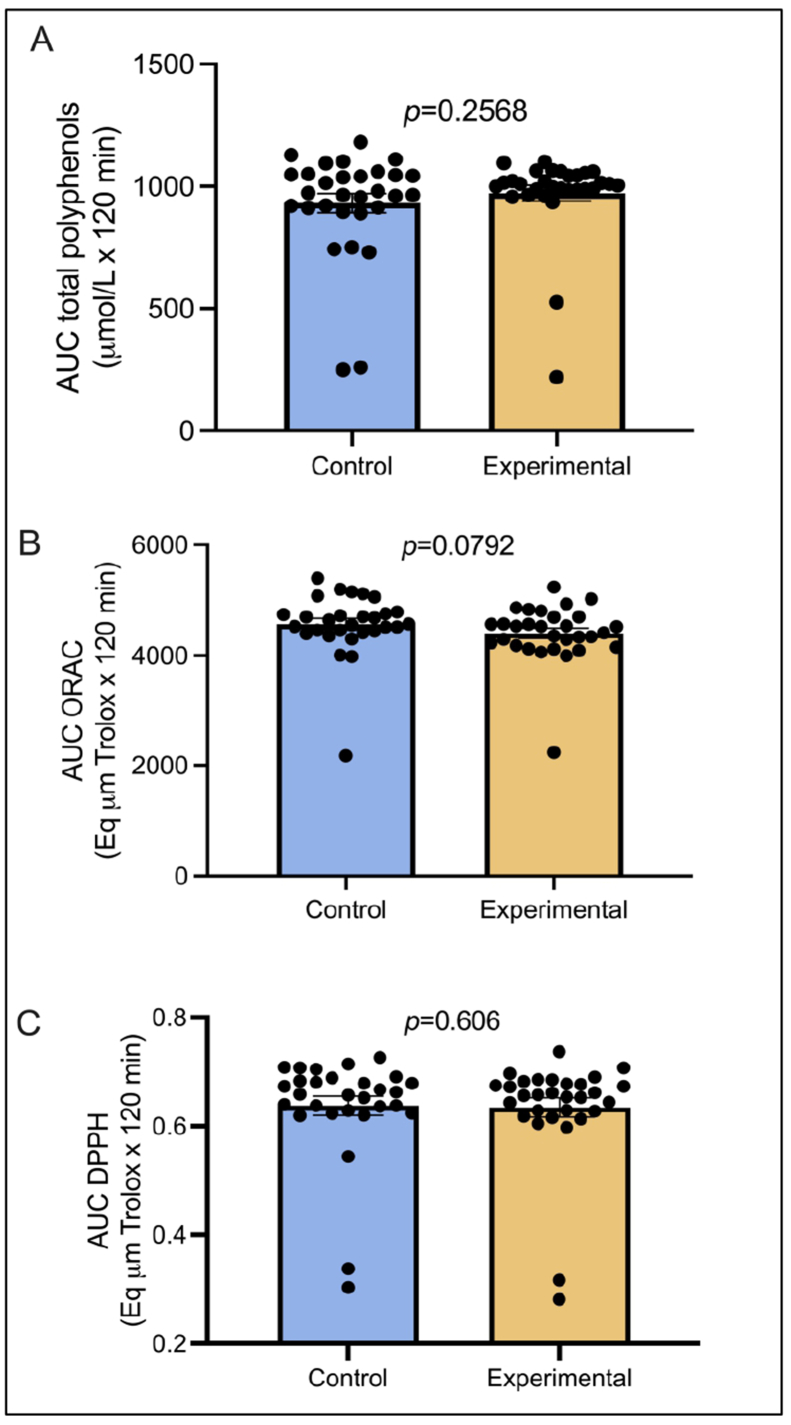# Pilot Trial of *Vachellia farnesiana* Pod Polyphenol Extract: Feasibility, Acute Postprandial Glycemic Response, and Incretin Hormone Modulation in Healthy Adults

**DOI:** 10.1016/j.cdnut.2026.107670

**Published:** 2026-03-12

**Authors:** Yonatan Y Cariño-Cervantes, Martha Guevara-Cruz, Omar Noel Medina-Campos, José Pedraza-Chaverri, Lilia Castillo-Martínez, Victoria Ramírez, Sara Montaño-Benavidez, Alexandro J Martagón, Paloma Almeda-Valdes, Luis Cisneros-Zevallos, Carlos A Aguilar-Salinas, Claudia Delgadillo-Puga

**Affiliations:** 1 Departamento de Nutrición Animal Dr. Fernando Pérez-Gil Romo, Instituto Nacional de Ciencias Médicas y Nutrición Salvador Zubirán, Ciudad de México, México; 2 Universidad Anáhuac, Maestría en Nutrición Clínica, Campus Norte, Estado de México, México; 3 Departamento de Fisiología de la Nutrición, Instituto Nacional de Nutrición y Ciencias Médicas Salvador Zubirán, Ciudad de México, México; 4 Departamento de Biología, Facultad de Química, Universidad Nacional Autónoma de México (UNAM), CDMX, México; 5 Servicio de Nutriología Clínica, Instituto Nacional de Ciencias Médicas y Nutrición Salvador Zubirán, Ciudad de México, México; 6 Unidad de Investigación de Enfermedades Metabólicas, Instituto Nacional de Ciencias Médicas y Nutrición Salvador Zubirán, Ciudad de México, México; 7 Departamento de Endocrinología y Metabolismo, Instituto Nacional de Ciencias Médicas y Nutrición Salvador Zubirán, Ciudad de México, México; 8 Department of Horticultural Sciences, Texas A&M University, College Station, TX, United States; 9 Dirección de Investigación, Instituto Nacional de Ciencias Médicas y Nutrición Salvador Zubirán, Ciudad de México, México

**Keywords:** safety, translational research, glucagon-like peptide-1, glucose-dependent insulinotropic polypeptide, glucose tolerance, *Vachellia farnesiana*

## Abstract

**Background:**

Medicinal plants contain bioactive compounds with potential benefits for metabolic regulation, including glucose homeostasis. *Vachellia farnesiana* (VF) pods are rich in polyphenols, including quercetin, catechins, methyl gallate, prutin, and hydroxycinnamic acids; however, their clinical effects in humans remain underexplored.

**Objectives:**

This study aimed to evaluate the feasibility, safety, and acute metabolic effects of a polyphenol-rich extract from VF pods in healthy adults, with emphasis on glycemic regulation and incretin hormone responses.

**Methods:**

A single-blind, randomized, controlled pilot trial was conducted in 60 healthy volunteers (BMI 18.5–24.9 kg/m^2^). Participants received a single oral dose of VF extract (1.2 mg/kg) or placebo (water) before a 75-g oral glucose tolerance test (OGTT). Blood glucose, insulin, glucagon-like peptide-1 (GLP-1), and glucose-dependent insulinotropic polypeptide (GIP) concentrations were measured at multiple time points. Safety was assessed through clinical symptoms, hepatic and renal biomarkers, and urinary levels of kidney injury molecule-1.

**Results:**

VF extract was well tolerated, with no adverse clinical events or biochemical changes in liver and kidney function. Glycemic reduction during OGTT (*P* = 0.117), as well as GLP-1 (*P* = 0.288) and GIP (*P* = 0.085) concentrations, did not reach statistical significance. Insulin concentrations remained unchanged, suggesting that incretin enhancement is independent of insulin stimulation.

**Conclusions:**

A single oral dose of VF polyphenol extract was safe for healthy individuals. In a predefined subsample (*n* = 10/group), incretin responses (GLP-1 and GIP) remained unchanged, suggesting that acute metabolic effects of VF are not mediated through incretin pathways. Although the extract produced only a modest effect on postprandial glycemia, this trial provides the first translational clinical evidence linking the ethnopharmacological use of VF with rigorously controlled human experimentation. These findings support its therapeutic promise in metabolic health, while underscoring the need for longer interventions, larger cohorts, and studies in individuals with impaired glucose regulation.

This trial was registered at clinicaltrial.gov as NCT05802472 (https://register.clinicaltrials.gov/prs/beta/studies/S000D2E700000124/protocol/protocolSummary).

## Introduction

Translational research plays a pivotal role in the evaluation, validation, and safety of ethnopharmacological knowledge in clinical practice. By integrating ethnopharmacological approaches with rigorous scientific standards inherent in translational research, we promote the development of safe, effective, and evidence-based medicinal plant-based therapies. These therapies hold promise as alternative interventions for metabolic dysfunctions associated with obesity [1]. In this context, an increasing number of studies have investigated bioactive compounds, particularly polyphenols, in plant-based foods and medicinal plants. These secondary metabolites have consistently demonstrated significant health-promoting properties, as substantiated by evidence from preclinical models, clinical trials, and epidemiological studies [2]. This highlights their therapeutic potential, particularly their modulatory effects on lipid metabolism and blood pressure regulation. Moreover, these compounds have been linked to enhanced insulin sensitivity and reduced systemic inflammation [3]. Furthermore, polyphenols have potential utility in the context of type 2 diabetes [4]. Despite these promising findings, further research on its bioavailability and bioactivity is crucial [5]. *Vachellia farnesiana* (VF), a Fabaceae shrub commonly known as “Huizache” in Mexico, is a notable source of these compounds. Polyphenols are highly concentrated in seeds, pods, and leaves. Traditionally, VF has been used in livestock and perfumery. The seeds and pods of VF are used in Mexican folk medicine to treat dysentery and tuberculosis and to relieve spasms and periodontal diseases [6,7]. In the past, we identified different compounds in VF, including flavonoids (e.g., quercetin, rutin, catechin, epicatechin, methyl gallate, naringenin, and prunin), hydroxycinnamic acids (e.g., p-coumaric, cinnamic, ferulic, and chlorogenic acids), and others [8]. Additionally, we reported the antioxidant activity and protection against oxidative-induced damage of VF pod extract in both *in vitro* and *in vivo* assays [9]. Other assays were conducted on animals to determine the anti-inflammatory properties of the VF pod extract, and we reported an important anti-inflammatory action compared with an anti-inflammatory medical drug [10]. On the basis of this evidence, we evaluated the effects of powdered VF pods or their polyphenol extract in mice fed a high-fat diet to assess whether this intervention could prevent the abnormalities induced by dietary obesity. Interestingly, we observed increased whole-body energy expenditure and mitochondrial activity in skeletal muscle, prevented insulin resistance, hepatic steatosis, and kidney damage, exerted immunomodulatory effects, and modified the fecal gut microbiota composition in mice fed a high-fat diet [11]. Additionally, we demonstrated the antitubercular and antidysentery activities of the isolated compounds from VF pods [8]. This evidence strongly suggests the potential health benefits of VF pods, offering hope for future medicinal research and applications. Additionally, the phytochemical polyphenol compounds have been shown to enhance the secretion of incretin hormones, such as glucagon-like peptide-1 (GLP-1) and glucose-dependent insulinotropic polypeptide (GIP). They also promote glucose-dependent insulin secretion and exert various beneficial effects beyond the classical incretin response [12]. This study aimed to evaluate the feasibility, safety, and acute metabolic effects of a polyphenol-rich extract from VF pods in healthy adults, with particular emphasis on glycemic regulation and incretin hormone responses.

## Methods

### Participants

This single-blind, randomized, controlled pilot trial enrolled 60 healthy adults aged 18–50 y with a BMI between 18.5 and 24.9 kg/m^2^. Eligibility criteria included normal fasting biochemical parameters: glucose (70–100 mg/dL), alkaline phosphatase (ALP) (44–147 U/L), alanine aminotransferase (ALT) (5–77 U/L), aspartate aminotransferase (AST) (8–33 U/L), total bilirubin (TB) (<1.2 mg/dL), indirect bilirubin (IB) (0.2–0.7 mg/dL), direct bilirubin (DB) (<0.3 mg/dL), and creatinine (0.6–1.3 mg/dL). The exclusion criteria were the presence of any chronic disease, use of dietary supplements, excessive alcohol or tobacco consumption, adherence to a prescribed diet, pregnancy, and breastfeeding.

Recruitment was conducted using posters and social media announcements authorized by the Communications Department of the Unidad de Enfermedades Metabólicas at the Instituto Nacional de Ciencias Médicas y Nutrición Salvador Zubirán (INCMNSZ). All participants received comprehensive oral and written information and provided signed informed consent before enrollment. The study was approved by the Research and Ethics Committees of the INCMNSZ (Ref. 3910) and was registered at clinicaltrials.gov (NCT05802472) and conducted in accordance with the Declaration of Helsinki and national data protection regulations. Recruitment occurred between 6 April, 2022 and 13 May, 2025.

### Study design

This was a single-blind, randomized, controlled clinical trial with a parallel-group design. The primary objective was to assess the acute feasibility, tolerability, and exploratory metabolic effects of a single oral dose of VF pod polyphenol extract in healthy adults. Participants were randomly assigned in a 1:1 ratio to the control or experimental group.

#### The control group received 250 mL of potable water

The experimental group received 250 mL of water containing a VF polyphenol extract (1.2 mg/kg, human-equivalent dose based on murine data).

#### Randomization and allocation concealment

Randomization was conducted using computer-generated blocks: 10 blocks of 6 and 1 block of 5, for a total of 11 blocks, to ensure balanced allocation [13]. The participants were randomly assigned to the control and experimental groups. An independent investigator prepared an allocation list to maintain allocation concealment. This approach offers several advantages over alternative methods. It employs a simple randomization technique to achieve proportional representation in the sample, prevent group imbalance, reduce variability, and enable more precise results than other methods [14].

#### Blinding procedures

To maintain blinding, the beverages were dispensed from opaque, sealed containers equipped with spouts. Sensory evaluation confirmed that the solution was indistinguishable from water in both taste and odor. All beverages were visually identical and differed solely by randomization code.

#### Study visits and procedures

The study protocol included 3 scheduled visits:

Visit 1 (screening) included an eligibility assessment, anthropometric measurements, fasting biochemistry, and informed consent.

Visit 2 (intervention) was conducted after an overnight fast of 10–12 h. Participants provided baseline blood samples, consumed the assigned beverage, and subsequently underwent an oral glucose tolerance test (OGTT; 75 g glucose). Serial blood samples were collected at 0, 20, 40, 60, and 120 min for analysis of glucose, insulin, incretin hormones (GLP-1, GIP), and serum antioxidant capacity.

Visit 3 (follow-up, 72 h postintervention) included evaluation of hepatic and renal function (AST, ALT, creatinine, and bilirubin) and measurement of urinary kidney injury molecule-1 (KIM-1) to assess renal safety.

All visits were conducted at the Unidad de Enfermedades Metabólicas, INCMNSZ.

### Plant materials

The pods of VF (L) Willd were meticulously collected in Acatlán de Osorio, Puebla, and Mexico, following strict geographical coordinates (parallels 18° 04'24" and 18° 21'30" north latitude, and the meridians 97° 55'18" and 98° 11'24" west longitude). A reference sample (Voucher number 8757) was deposited in the Herbarium of FES-Cuautitlán, Universidad Nacional Autónoma de México, ensuring the traceability and authenticity of our research. The plant name has been cross-verified with the Plant List (http://www.theplantlist.org). We adhered to the international, national, and institutional rules concerning biodiversity rights, further reinforcing the credibility of our findings.

#### VF extraction and total polyphenol content

VF was collected, and its botanical identification was performed when dried whole fruits of VF were ground and mixed in an 80:20 methanol–water solution. The extraction was performed as described by Delgadillo-Puga et al. [9], and the total polyphenol content (TPC) was determined using the method proposed by Singleton et al. [15]. Additionally, nutrients analysis was reported (Table 1) Importantly, the same batch of VF pods used in this clinical trial had been previously characterized in detail by liquid chromatography-mass spectrometry (LC–MS) and nuclear magnetic resonance (NMR) in our earlier publications by Hernández et al. [8] and Delgadillo-Puga et al. [9], confirming a diverse phenolic profile such as methyl gallate, gallic acid and (2S)-naringenin 7-O-β-D-glucopyranoside. This ensures consistency and traceability of the extract employed.

**TABLE 1:** Polyphenols and nutrients (g/100 g) of the *Vachellia farnesiana* extract.

Components	
Dry matter	96.6
Protein	4.7
Fat	5.8
Ash	2.3
Energy (kcal/100 g)	42.96
TPC gallic acid equivalents (GAE) (g/100 g DM)	7.82

Abbreviation: TPC, total polyphenol content; DM, Dry matter.

#### Calculation of human equivalent dose

The equivalent human dose was determined based on a preclinical study of Delgadillo-Puga et al. [11] conducted by this research team over several years. To establish a safe human dose in murine models, no observed adverse effect concentrations were considered. A dose of 10 mg/kg of VF extract, previously reported to be safe in murine models, was selected. Conversion from murine to human dose was performed using formulas from the Food and Drug Administration (FDA) [16] and Nair and Jacob [17] to obtain Equation (1). Additionally, the human equivalent dose (HED) was calculated based on the body surface area (BSA, m^2^) normalization. Initial estimates were based on a reference individual (60 kg and 170 cm), with final adjustments made according to each participant's anthropometric data. BSA was determined using the Shuter and Aslani formula [18], as described in Equations (2) and (3). The FDA [17] was applied at 10% of the calculated HED to minimize risks in this early phase [Equation (4)], with 90% remaining for future exploration.

**Equation 1.** Calculation of the HED

$$\text{HED}=\text{animal}\;\text{dose}\;\left(\text{mg}/\text{kg}\right)\times \left(\frac{Animal\;km}{Human\;km}\right)=\text{human}\;\text{dose}\;\left(\text{mg}/\text{kg}\right)$$

**Equation 2.** Correction factor

$$\text{Factor}\;\text{human}\;\text{or}\;\text{animal}\;K_{m}=\left(\frac{Weight\;\left(\text{kg}\right)}{BSA\;\left(m^{2}\right)}\right)=\text{kg}/m^{2}$$

**Equation 3.** BSA calculation

$$\text{BSA}m^{2}=\left\{\left[\left(\text{Weight}{\left(\text{kg}\right)}^{0.425}\right)\left(\text{Height}{\left(\text{cm}\right)}^{0.725}\right)\right]\right\}\times 0.007184=m^{2}$$

**Equation 4.** Final HED calculation

$$\text{HED}=10\times \left[\frac{4.29\left(Animal\;km\right)}{35.5\left(Human\;km\right)}\right]=1.2\;\text{mg}/\text{kg}$$

### Biological sample collection and analyses

#### Glycemic and insulin response

During visit 2, scheduled within 7 d of visit 1, participants were randomly assigned to the experimental (*n* = 30) or control groups (*n* = 30). Following an 8–10-h overnight fast, a peripheral venous catheter was inserted using standardized nursing protocols for central blood sampling. A baseline blood sample (time 0) was obtained to measure the fasting glucose and insulin concentrations. Subsequently, the experimental group received 250 mL of potable water containing the VF extract, whereas the control group received 250 mL of water alone. Fifteen minutes after administration, the participants underwent an OGTT, which involved the ingestion of 75 g of monohydrated glucose. Blood glucose and insulin concentrations were measured at 20, 40, 60, and 120 min after glucose loading. After the OGTT, the catheter was removed using an aseptic technique. Serum samples were collected at 0, 60, and 120 min to determine the concentrations of GIP and GLP-1. To estimate dietary polyphenol intake, a food frequency questionnaire was adapted by the Instituto Nacional de Salud Pública. Reported food items were analyzed using the Phenol Explorer 3.0 database by Rothwell et al. [19], considering cooked foods and expressing polyphenol content per 100 g or mL of food. Urine samples were collected at the end of the experiment.

#### Safety assessment and biomarkers profiling of hepatic and renal function

Safety monitoring was performed immediately after the OGTT, using an adverse event questionnaire. This questionnaire comprised closed-ended questions designed to evaluate clinical signs and symptoms, including nausea, vomiting, gastrointestinal pain, dizziness, headache, and other potential adverse effects. Collected data were recorded and stored in a database on the RedCap platform. Visit 3 was conducted 72 h after visit 2 to specifically assess delayed adverse effects. During this visit, fasting blood and urine samples were collected, a second adverse event questionnaire was administered, and a 24-h dietary recall was conducted to evaluate caloric intake, as no nutritional restrictions were imposed. Target organs for adverse effects were the liver and kidneys. Accordingly, biomarkers such as AST, ALT, ALP, TB, DB, IB, and creatinine were measured both before and 72 h after experimental beverage administration. The referral thresholds for these markers were defined as AST >96 U/L, ALT >99 U/L, ALP >150 U/L, and creatinine >1.5 mg/dL. Urine samples were analyzed for acute KIM-1 using an ELISA kit (ENZO, Cat. ADI-900-224) at a 1:4 dilution. KIM-1 concentrations were normalized to creatinine and expressed as a ratio per urine volume. Acute kidney injury was subsequently defined as a KIM-1 concentration exceeding 300 pg/min.

#### Quantification of biochemical and hormonal parameters

Blood samples were collected, immediately centrifuged, aliquoted into cryovials labeled with a unique identification number and collection date to ensure traceability and preservation, and then stored at −80°C. Hepatic profile, specifically AST concentrations (cat. 442665), ALT (cat. 442620), and ALP (cat. 442670) concentrations, were measured using the Beckman Coulter Unicel DxC 600 Synchron Clinical System. Creatinine concentrations were measured using a commercial kit, QuatiChrom Creatinine Assay Kit (Bioassays System), according to the manufacturer's instructions. Insulin concentrations (cat. 33410) were quantified separately using the Beckman Coulter Access 2. For the quantification of GIP and GLP-1 active, a Milliplex Metabolic Assay (HMNH3-34K, Millipore, using magnetic regions 21 and 22, respectively) was employed based on magnetic microspheres and the Luminex xMAP detection method. Samples were collected in tubes pretreated with dipeptidyl peptidase IV (DPP-IV) inhibitor to prevent hormone degradation. All samples were handled under a strict cold chain protocol, with all processing steps (including centrifugation) adhering to manufacturer recommendations and established laboratory standards, and were stored at −20°C until analysis.

**FIGURE 1:**
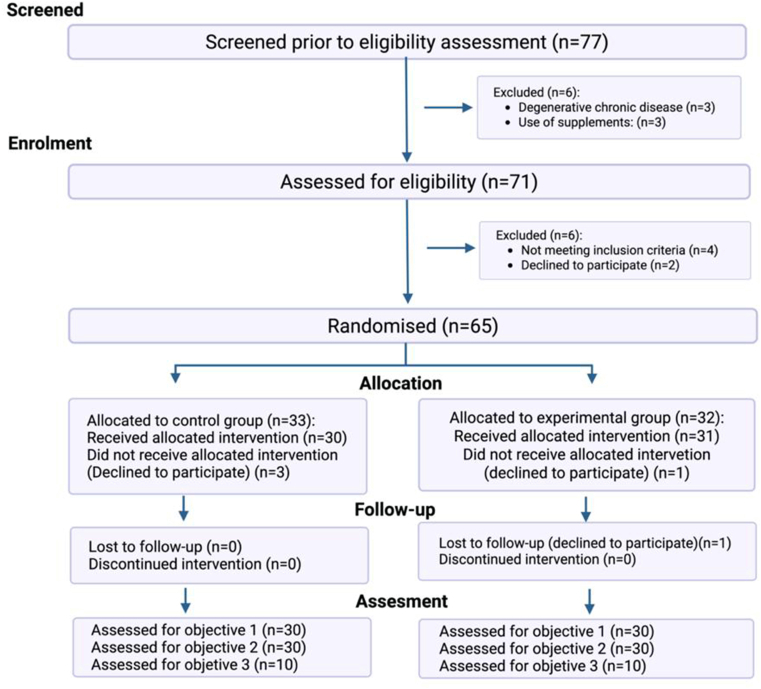
CONSORT flow diagram, which outlines participant screening, eligibility assessment, randomization, allocation, follow-up, and analysis. A total of 77 individuals were screened, 71 met eligibility criteria, and 65 were randomized to the control or experimental group. Sixty participants completed the intervention and were assessed according to the predefined study objectives: objective 1 (safety evaluation), objective 2 (postglucose challenge glycemic and insulin response), and objective 3 (incretin hormone response). All enrolled participants were healthy adults with normal baseline biochemical parameters.

Incretin hormones, specifically GLP-1 and GIP, were measured in a predefined subsample of 10 participants per group. Safety biomarkers, glucose, and insulin responses were evaluated in the entire cohort of 30 participants per group (Figure 1).

#### Total phenolic content and antioxidant activity in blood serum

The TPC of each sample was determined using the Folin–Ciocalteu reagent (FCR) as follows: samples were deproteinized by mixing 50 μL of blood serum with 25 μL of 20% metaphosphoric acid, followed by centrifugation at 5000 × g for 5 min, according to Koshiishi et al. [20]. The resulting supernatant was then used for the TPC assay, adapted from the method described by Álvarez et al. [21]. In a 1.5 mL microcentrifuge tube, 20 μL of deproteinized blood serum was mixed with 15 μL of 10% FCR and incubated at room temperature for 5 min. Subsequently, 50 μL of 20% sodium carbonate and 150 μL of distilled water were added. The mixture was then incubated for 20 min and centrifuged at 4000 × *g* for 4 min. Finally, 180 μL of supernatant was transferred to a 96-well microplate (Costar cat. 2595) and the optical density at 735 nm was measured using a Synergy HT multimode microplate reader (BioTek Instruments, Inc.). The results were expressed as gallic acid equivalents (GAE). The oxygen radical absorbance capacity (ORAC) assays were performed according to the method described by Huang et al. [22], using 2,2'-azobis(2-methylpropionamidine) dihydrochloride (AAPH) as the peroxyl radical generator, Trolox as the standard, and fluorescein as the fluorescent probe. Briefly, the fluorescence of a reaction mixture containing 25 μL of water (blank), Trolox standards, or diluted samples (1:400), along with 25 μL of 153 mM AAPH and 150 μL of 50 nM fluorescein, was measured every minute for 90 min at 37°C. Measurements were taken at an excitation wavelength of 485 nm and an emission wavelength of 520 nm using a Synergy HT multi-mode microplate reader (BioTek Instruments, Inc.). ORAC values were calculated from the net area under the decay curves and were expressed as μmoles of Trolox equivalents. The 2,2-diphenyl-1-picrylhydrazyl (DPPH) radical scavenging activity was evaluated according to the method described by Koren et al. [23]. Briefly, 35 μL of the 1:2 diluted sample was mixed with 25 μL of a 2 mM DPPH solution in a 1.5 mL centrifuge tube. After a 2-min incubation period, 400 μL of ethanol was added, and the resulting mixture was incubated for an additional 2 min at room temperature. Following centrifugation at 2000 × *g* for 2 min, the optical density of the supernatant was measured at 517 nm using a 96-well microplate. Antioxidant capacity was expressed as mM equivalents of Trolox.

### Statistical analysis

To determine the sample size, a 2-tailed comparison formula was applied, setting a confidence level of 95% (1 alpha), with a statistical power of 80% and accounting for variation (s), particularly in blood glucose concentration and the target difference to be detected (d) (*s* = 193 mg/dL and *d* =154 mg/dL, respectively), based on theoretical precedents. A dropout rate of 20% was factored in, using the following formula:

$n=\left(\frac{2{\left(1.960+0.842\right)}^{2}\times {\left(193\right)}^{2}}{{\left(154\right)}^{2}}\right)=$24.6 + 20%= 30 participants per group.

All statistical analyses were performed using Jamovi software (version 2.3.2.0; The Jamovi Project), and figures were generated using GraphPad Prism (version 7.0; GraphPad Software). Continuous variables were reported as mean ± SD or median with IQR, according to the distribution evaluated using the Shapiro–Wilk test for normality. Based on the distribution, either Student’s *t*-test or the Mann–Whitney *U* test was used for AUC group comparisons of total polyphenols and antioxidant activity. To estimate the difference between time-by-group comparisons for the intervention's effect on postintervention outcomes, controlling for clinical baseline differences, an analysis of covariance of repeated measures was conducted, followed by a Bonferroni post-hoc test for clinic safety monitoring. The dependent variable was the postintervention measurement; the fixed factor was treatment group (control, experimental); the corresponding baseline value was used as a covariate, as were variables with clinical baseline differences (ALT, TB, and HOMA-IR). Analysis of covariance models were used to compare the AUC of glucose and insulin, controlling for clinical baseline differences (ALT, TB, and HOMA-IR), and results were reported as estimated marginal means with corresponding SEs and 95% confidence intervals, adjusted for the specified covariates as mentioned. All analyses were done per-protocol. Statistical significance was defined as *P* < 0.05.

## Results

### Recruitment and clinical demographic characteristics of the participants

Of the 77 healthy participants initially assessed, 12 were excluded for not meeting the inclusion criteria (specifically, abnormal glucose concentrations and elevated liver enzymes beyond the established threshold). The remaining 65 participants were randomly assigned to the 2 groups. During the follow-up period, 5 participants were lost to follow-up, resulting in a final analytical sample of 60 individuals. The participant flow diagram is presented in Figure 1 and was developed in accordance with the CONSORT guidelines described by Eldridge et al. [24]. The study population consisted of healthy adults (mean age: 28 y; BMI: 23.5 kg/m^2^), predominantly female (61.7%). All participants exhibited biochemical parameters within normal ranges and no evidence of metabolic disorders. No differences were found between groups in baseline characteristics, including anthropometrics, vital signs, caloric intake (∼2100 kcal/d), macronutrient distribution, and total polyphenol intake. Although the primary source of polyphenols was fruits and the lowest was legumes, this consumption pattern was homogeneous across groups (Table 2).

**TABLE 2:** Anthropometric, clinical, and dietary baseline characteristics of healthy volunteers in each group.

Variable	Control	Experimental
Women% (*n*)	56.6 (17)	66.6 (20)
Men% (*n*)	43.4 (13)	33.4 (10)
Age (y)	28 (22.3–35.3)	28 (24–34)
Weight (kg)	66.7 (57.7–74.4)	60.5 (56.9–75.6)
BMI (kg/m^2^)	24.1 (22.2–26.9)	23.0 (21.9–24.9)
Fat mass (%)	28.1 (22.4–33.3)	29.5 (25.8–33.2)
Free fat mass (%)	71.9 (66.7–77.6)	71.0 (67.6–74.7)
Waist circumference (cm)		
Women	75.5 (70.5–81)	74.4 (70.7–81.1)
Men	84 (82.5–90.7)	82.3(75.3–88.3)
Hip circumference (cm)		
Women	97.9 ± 6.3	95.9 ± 6.4
Men	97.8 ± 6.8	96.6 ± 6.4
Waist/Hip Index	0.82 ± 0.07	0.82 ± 0.07
SBP (mmHg)	107 (101–114)	105 (97.8–112)
DBP (mmHg)	69.6 ± 6.5	67.2 ± 7.0
Heart rate (bpm)	73.0 ± 9.3	70.9 ± 11.7
Fasting glucose (mg/dL)	90.9 ± 7.5	87.9 ± 7.2
HOMA-IR	1.7 ± 1.0	1.2 ± 0.6
AST (U/L)	20.5 (18.0–26.2)	21.0 (19.0–24.0)
ALT (U/L)	22.0 (15.5–27.2)	17.5 (15.2–26.2)
ALP (U/L)	50 (40.7–63.0)	49.5 (44.2–60.0)
IB (mg/dL)	0.7 (0.5–0.8)	0.6 (0.4–0.7)
DB (mg/dL)	0.1 (0.1–0.2)	0.1 (0.1–0.2)
TB (mg/dL)	0.8 (0.6–1.0)	0.7 (0.5–0.8)
Serum creatinine (mg/dL)		
Women	0.7 ± 0.1	0.7 ± 0.1
Men	0.9 (0.8–1.0)	0.9 (0.8–1.0)
Energy (kcal)	2112.3 ± 748.7	2237.3 ± 859.8
Carbohydrate distribution (%)	48.8 (39.5–51.6)	43.2 (41.5–47.9)
Protein (g/kg/d)	1.3 (1.29–1.9)	1.4 (1.0–2.18)
Protein distribution (%)	19.9 (16–25.4)	18.5 (13.9–22.8)
Fat distribution (%)	34.9 (30.4–39.0)	34.1 (30.4–43.1)
Polyphenol (mg/d)	273.8 ± 197.7	280 ± 214.8

Sample size: *n* = 30 per group. Data were presented as mean ± SEM or median with IQR (25th and 75th percentiles). Sample size: *n* = 30 per group.

Abbreviations: ALP, alkaline phosphatase; ALT, alanine aminotransferase; AST, aspartate aminotransferase; DB, direct bilirubin; DBP, diastolic blood pressure; IB, indirect bilirubin; SBP, systolic blood pressure; TB, total bilirubin.

### No adverse effects were observed with experimental beverage intake

Overall, no adverse effects directly attributable to the intake of the experimental beverage were observed. Analysis of the closed-ended adverse event questionnaire data revealed no statistically significant differences in the reported symptoms between groups. The control exhibited a higher prevalence of symptoms, such as dizziness, nausea, and headache, which may be attributed to prolonged fasting at the end of visit 2 and/or the ingested glycemic load; these symptoms resolved after food intake. To confirm the nonpersistence of effects, a second questionnaire was administered 72 h after beverage consumption. In this follow-up, the control group reported headaches, whereas only 1 subject in the experimental group reported this symptom, along with nausea. Nevertheless, all reported symptoms were considered nonspecific (Table 3). Further safety monitoring included the evaluation of KIM-1, an indicator of acute kidney injury, measured 3 h after experimental beverage consumption. KIM-1 concentrations did not increase in any groups, and values in both groups did not exceed 300 pg/min, indicating the absence of acute kidney injury. Furthermore, serum results for liver or renal function enzymes or markers showed no elevations suggestive of damage, as detailed in Table 3, which presents baseline and postadministration results.

**TABLE 3:** Clinic safety monitoring after control and experimental beverage intake in participants.

Variable	Control		Experimental		*P* value^1^
	Visit 1	Visit 3	Visit 1	Visit 3	
ALP (U/L)	52.1 ± 2.29	51 ± 2.26	50.9 ± 2.33	47.9 ± 2.31	0.29
ALT (U/L)	24.5 ± 2.32	21.8 ± 1.8	22 ± 2.37	21.4 ± 1.83	0.35
AST (U/L)	22.3 ± 0.82	21.5 ± 1.1	22.3 ± 0.84	22.9 ± 1.1	0.38
TB (mg/dL)	0.92 ± 0.1	0.71 ± 0.1	0.80 ± 0.1	0.6 4 ± 0.1	0.43
IB (mg/dL)	0.70 ± 0.01	0.59 ± 0.04	0.68 ± 0.01	0.57 ± 0.04	0.95
DB (mg/dL)	0.13 ± 0.02	0.14 ± 0.01	0.16 ± 0.02	0.15 ± 0.01	0.41
Serum creatinine (mg/dL)	0.81 ± 0.03	0.82 ± 0.03	0.82 ± 0.03	0.81 ± 0.03	0.31

Adverse effects	Control		Experimental	
	Visit 1 *n* (%)	Visit 3 *n* (%)	Visit 1 *n* (%)	Visit 3 *n* (%)
Headache	4 (13.3)	2 (6.6)	0 (—)	1 (3.3)
Dizziness	2 (6.6)	0 (—)	1 (3.3)	0 (—)
Nausea	1 (3.3)	0 (—)	1 (3.3)	0 (—)
Gastrointestinal irritation	1 (3.3)	0 (—)	0 (—)	0 (—)
Vomiting	0 (-)	0 (—)	0 (—)	0 (—)
Gastrointestinal pain	0 (-)	0 (—)	0 (—)	0 (—)
Other	0 (-)	0 (—)	0 (—)	0 (—)

Urine biomarker KIM-1	Visit 3		*P*-value	
Control	70.88 (31.6–131)		0.333	
Experimental	99.70 (60.6–162)			

Data were presented as mean ± SEM or median with IQR (25th and 75th percentiles). Sample size: *n* = 30 per group.

Abbreviations: ALP, alkaline phosphatase; ALT, alanine aminotransferase; AST, aspartate aminotransferase; DB, direct bilirubin; IB, indirect bilirubin; KIM-1, kidney injury molecule-1 (pg/min); TB, total bilirubin.

1 Between-group comparisons at 72 h postintervention (visit 3) were analyzed using analysis of covariance (ANCOVA). Postintervention values of hepatic and renal biomarkers were entered as dependent variables; the treatment group (control vs. experimental) was treated as a fixed factor; and corresponding baseline (visit 1) values (ALT, TB, and HOMA-IR) were included as covariates.

### VF extract induces moderate glycemic reduction and enhanced incretin secretion in healthy participants

**FIGURE 2:**
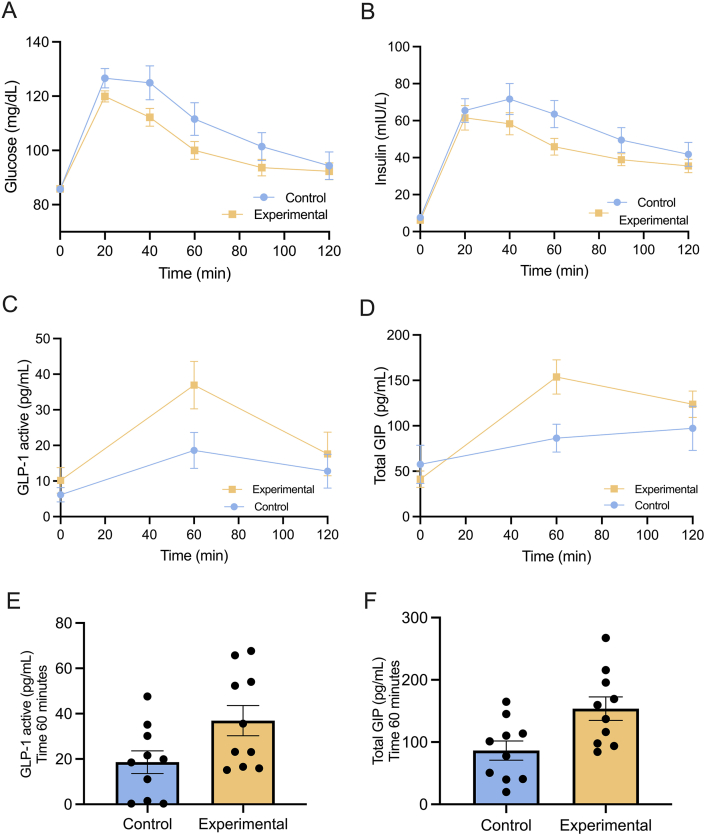
Glycemic modulation and hormonal effect of the *Vachellia farnesiana* (VF) extract in healthy participants. (A) Serum glucose concentration curve in healthy participants, (B) serum insulin concentration curve in healthy participants, and (C) serum concentration curve of glucagon-like peptide-1 (GLP-1); values at the 60-min mark are presented in a graph with an independent samples t-test statistical analysis. (D) Serum concentration curve of glucose-dependent insulinotropic polypeptide (GIP). (E) Concentrations of GLP-1 active at the 60-min mark are presented in a graph with an independent sample t-test statistical analysis. (F) Concentrations of GIP at the 60-min mark are presented in a graph with an independent sample t-test statistical analysis. Data are presented as the means ± SEM. A and B (control, *n* = 30 and experimental, *n* = 30), and C and D (control, *n* = 10 and experimental, *n* = 10). ∗Statistical significance at *P* < 0.05.

The present study evaluated the glycemic response following administration of a single dose of VF extract. The results indicated that the intake of the VF extract beverage induced a moderate reduction in blood glucose concentrations in the experimental group compared with the control. The lowest glucose concentrations were recorded between 40 and 60 min during the OGTT (Figure 2A). However, no statistically significant differences were observed in the area under the curve (AUC mg/dL × 120 min) between the experimental and the control, with values of 217 (184.7–243.2) and 198.3 (186.3–219.3), respectively, (*P* = 0.088), as detailed in Table 4.

**TABLE 4:** Glucose, insulin, and incretin area under the curve in healthy participants.

	Control	Experimental	*P* values^1^
Glucose AUC (mg/dL × 120 min)	216 ± 6.58	201 ± 6.46	0.117
Glucose iAUC (mg/min/dL)	46.1 ± 6.43	29.5 ± 6.31	0.080
insulin AUC (mg/dL × 120 min)	10.6.4 ± 7.86	88.8 ± 7.71	0.128
insulin iAUC (mg/min/dL)	92.5 ± 7.93	75.3 ± 7.79	0.139
GLP-1 AUC (pg/mL × 120 min)	36.9 ± 9.15	52.3 ± 8.51	0.288
Total GIP AUC (pg/mL × 120 min)	200 ± 47.1	334 ± 43.8	0.085

Data were presented as mean ± SEM. Sample size: *n* = 30 per group for glucose and insulin; *n* = 10 per group for incretin analyses (GLP-1, GIP).

Abbreviations: ALT, alanine aminotransferase; ANCOVA, analysis of covariance; iAUC, incremental AUC; GIP, glucose-dependent insulinotropic polypeptide; GLP-1, glucagon-like peptide-1.

1 Adjusted by ALT, TB (total bilirubin), and HOMA-IR as covariates used ANCOVA analysis. Data were presented as estimated marginal means ± with SEs.

To further investigate the moderate hypoglycemic effects observed during the OGTT and determine whether an increase in insulin synthesis mediated these effects, insulin concentrations were quantified. Contrary to expectations, the experimental group exhibited slightly lower insulin concentrations than the control throughout the OGTT curve, with no statistically significant differences in the insulin AUC (*P* = 0.117). This phenomenon may be attributed to the fact that clinically healthy participants-maintained glucose homeostasis mechanisms that compensated for the reduction in glucose concentrations, thereby preventing a drastic decrease in blood glucose concentrations (Figure 2B). Additionally, it has been reported that specific polyphenols, such as those present in the VF extract, can modulate the activity of incretins (GLP-1 and GIP; Figure 2C and D), which play an essential role in regulating glucose metabolism. To explore this mechanism, serum concentrations of these hormones were measured. The results did not demonstrate a statistically significant increase in the concentrations of active GLP-1 (Figure 2E) and total GIP (Figure 2F) in the experimental group compared with the control group (*P* = 0.288 and *P* = 0.085, respectively).

### Serum polyphenols and antioxidant activity remain at baseline concentrations despite confirmed total polyphenol content in the extract of the VF sample

The quantification of total polyphenol content in the methanolic extract of the VF sample, determined by spectrophotometry at 750 nm using the Folin–Ciocalteu method, revealed a concentration of 7.82 g of GAE per 100 g of dry extract (dry weight). Serum samples collected were analyzed in control and experimental (visit 2) at baseline (0 min) and at 20, 40, 60, and 120 min after consumption of either the experimental beverage or the control beverage. No significant differences were observed in the AUC between groups. Similarly, antioxidant capacity AUC (ORAC/DPPH) showed no statistically significant intergroup (Supplemental Figure 1).

## Discussion

This randomized, single-blind, controlled pilot feasibility trial investigated the safety and acute metabolic effects of a single oral dose of a polyphenol-rich extract from VF pods in healthy volunteers. Although the overall glycemic AUC during the postoral glucose tolerance test OGTT did not achieve statistical significance (*P* = 0.088), the observed postprandial glucose reduction between 40 and 60 min indicated a physiologically relevant trend, likely moderated by the robust glucose homeostasis mechanisms inherent in healthy individuals. This observation aligns with the known properties of plant polyphenols, which can modulate carbohydrate metabolism by inhibiting digestive enzymes and enhancing peripheral glucose uptake and insulin signaling pathways [9,25]. Contrary to our initial hypothesis, the extract did not alter circulating incretin concentrations (GLP-1 or GIP). Measurements were collected from a predefined subsample of 10 participants per group, limiting statistical power to detect subtle hormonal differences. Under the acute glucose-challenge conditions applied in this study, incretin secretion remained unchanged. These findings suggest that the metabolic effects associated with VF may involve mechanisms beyond incretin-mediated pathways. These incretinotropic effects occurred without significant changes in systemic insulin concentrations, suggesting improved insulin efficiency or involvement of noninsulin-mediated glucose disposal pathways. Previous evidence for incretin modulation by *Vachellia* species has been limited to preclinical studies involving related taxa. For instance, polysaccharides from *Acacia tortilis* have been shown to significantly increase GLP-1 concentrations in diabetic rats [26], and *Acacia arabica* extracts have been found to modulate GLP-1 and GIP sensitivity in murine models [27]. The phenolic compounds present in VF, such as methyl gallate, naringenin, and catechins, may stimulate incretin secretion or inhibit DPP-IV, thereby enhancing the bioavailability and activity of incretin [28]. The VF extract also exhibited a favorable acute safety profile. No adverse effects were reported, nor were any clinically significant changes observed in hepatic or renal biomarkers, including AST, ALT, ALP, bilirubin, creatinine, and the early renal injury marker KIM-1. These findings align with previous toxicological studies in murine models, which showed that oral doses of up to 10 mg/kg of VF extract were nontoxic and did not induce histopathological or biochemical alterations [11]. Additionally, our in vitro studies using renal epithelial cells have demonstrated the antioxidant and cytoprotective properties of polyphenols derived from *Acacia farnesiana* [19]. The phytochemical composition of the VF extract employed in this study was thoroughly characterized in prior work using LC–MS and NMR techniques, confirming a diverse profile of bioactive phenolics [8]. These compounds are known to modulate oxidative stress and inflammation, both of which are closely linked to glucose metabolism and incretin signaling [29,30]. The VF polyphenol-rich extract demonstrated a total phenolic content of 7.82 g GAE per 100 g dry matter. This value is substantially higher than that of raspberry leaf tea, which contains ∼0.5 g polyphenols per 100 g, as reported by Alkhudaydi and Spencer [31]. Therefore, the extract used in this study provides a total polyphenol content nearly 15 times that of the raspberry-based intervention. Alkhudaydi and Spencer [31] also investigated acute postprandial responses to sucrose and found that raspberry polyphenols reduced early glucose and insulin peaks by inhibiting α-glucosidase and β-fructofuranosidase. In contrast, the present findings indicate that VF polyphenols, even at substantially elevated concentrations, do not induce hepatotoxic or nephrotoxic effects, thereby supporting their potential as safe bioactive ingredients. These effects warrant further investigation using metabolic challenge models such as oral sucrose tolerance tests. Future studies should evaluate the effects of chronic administration and include individuals with prediabetes or type 2 diabetes mellitus to determine the therapeutic relevance of the VF extract under pathophysiological conditions.

### Limitations

Incretin hormone analysis was conducted in a limited subsample (*n* = 10 per group), restricting statistical power. The single-dose design in healthy participants also limits generalizability to populations with metabolic impairments. Additionally, although this study only quantified TPC using the Folin–Ciocalteu method, we emphasize that the batch used was the same as that previously profiled by LC-MS/NMR, ensuring that the bioactive composition was robustly established. Another limitation of this study was the lack of a washout period and dietary control for polyphenol intake. However, a food frequency questionnaire was used to estimate baseline consumption. The study followed a per-protocol analytical approach, as only participants who completed the intervention (*n* = 60) were included in the analysis. Because an intention-to-treat strategy was not planned in the original design, the results may be less generalizable and potentially more sensitive to attrition-related biases. Additionally, the single-blind design, in which participants were blinded, but investigators were not, may have introduced performance or measurement bias. Future studies should use a double-blind, intention-to-treat design to minimize these potential sources of bias.

In summary, this pilot randomized controlled trial offers initial clinical evidence that a single oral dose of a polyphenol-rich extract from VF pods is safe for healthy individuals. Although the extract produced a moderate reduction in postprandial glycemia, it did not increase the secretion of the incretin hormones GLP-1 and GIP. These outcomes occurred without adverse clinical symptoms or biochemical changes in hepatic or renal function. Notably, this study is the first to translate ethnopharmacological knowledge of *VF* into controlled human experimentation, thereby supporting its therapeutic potential for metabolic health. However, additional research with more extended administration periods, larger cohorts, and participants with impaired glucose regulation is necessary to confirm and extend these preliminary results.

## Author contributions

The authors’ responsibilities were as follows – YYC-C, CD-P, MG-C, ONM-C, JP-C, VR, SM-B, PA-V: contributed to writing – original draft, methodology, and conceptualization; YYC-C, CD-P: contributed to funding acquisition, validation, and investigation; YYC-C, CD-P, AJM, CAA-S, LC-Z: contributed to writing – review & editing and methodology; and all authors: read and approved the manuscript.

## Data availability

The original data in this study are presented in this article. For further inquiries, contact the corresponding authors. Data sharing: https://docs.google.com/spreadsheets/d/1805eRqqZuC6s0F6N8k5KxO6P2z8tjDsb6BsaPnYH-M0/edit?gid=0#gid=0

## Funding

The work was partially supported by the Fund to Support Research Project in Health 2023, by the Instituto Nacional de Ciencias Médicas y Nutrición Salvador Zubirán, and by Programa de Apoyo a Proyectos de Investigación e Innovación Tecnológica (PAPIIT)-UNAM No. IN202725.

## Declaration of generative AI and AI-assisted technologies in the writing process

During the preparation of this manuscript, Grammarly was used solely for language editing purposes. No AI tools were used to generate or modify scientific content, data interpretation, or conclusions. The author assumes full responsibility for the content of this manuscript.

## Conflict of interest

The authors declare that they have no known competing financial interests or personal relationships that could have appeared to influence the work reported in this paper.

## Figures and Tables

**Figure:**